# Editorial: Use of the RE-AIM Framework: Translating Research to Practice With Novel Applications and Emerging Directions

**DOI:** 10.3389/fpubh.2021.691526

**Published:** 2021-06-11

**Authors:** Paul A. Estabrooks, Bridget Gaglio, Russell E. Glasgow, Samantha M. Harden, Marcia G. Ory, Borsika Adrienn Rabin, Matthew Lee Smith

**Affiliations:** ^1^Department of Health Promotion, University of Nebraska Medical Center, College of Public Health, Omaha, NE, United States; ^2^Clinical Effectiveness and Decision Science, Patient-Centered Outcomes Research Institute, Washington, DC, United States; ^3^Dissemination and Implementation Science Program of Adult & Child Consortium for Health Outcomes Research & Delivery Science (ACCORDS), Department of Family Medicine, School of Medicine, University of Colorado, Aurora, CO, United States; ^4^Physical Activity Research and Community Implementation, Human Nutrition, Foods, and Exercise, Virginia Tech, Blacksburg, VA, United States; ^5^Center for Population Health and Aging, Texas A&M University, College Station, TX, United States; ^6^Department of Environmental and Occupational Health, School of Public Health, Texas A&M University, College Station, TX, United States; ^7^Herbert Wertheim School of Public Health and Human Longevity Science, UC San Diego, San Diego, CA, United States

**Keywords:** RE-AIM, planning, evaluation, dissemination, implementation, research, practice

## Introduction

In 2019, we initiated a call for papers to contribute to a Frontiers of Public Health Research Topic on the use of the Reach, Effectiveness, Adoption, Implementation, and Maintenance (RE-AIM) Framework. In part, the Research Topic was intended to celebrate 20 years of planning and evaluation using RE-AIM and underscore new research directions. More importantly, we saw the need to document how the framework continues to be adapted, evolve, and provide opportunities in emerging areas. Of key importance is speeding research-practice translation and studying the process that leads to more efficient movement of evidence-based principles, programs, and policies into sustained community and clinical practice. What follows is an overview of the excellent submissions we received that document lessons learned, adaptations, and innovative uses of RE-AIM, as well as highlight the framework's potential to advance science, quality of practice, and population health.

The Research Topic and resulting papers are grouped into four sections: (a) *Introduction*; (b) *Use of RE-AIM in Community Settings*; (c) Use of RE-AIM in Clinical Settings; and (d) *Emerging Directions in the Application of RE-AIM*. The *Introduction* section kicks off the collection with a paper led, fittingly, by (Glasgow, Harden et al.), on how the RE-AIM Framework has evolved over the 20 years since the seminal American Journal of Public Health (Glasgow, Vogt et al.) article was published in 1999. This is accompanied by an insightful commentary from Dr. Kurt Stange that highlights the value of using RE-AIM and its contextual extension, the Practical, Robust, Implementation, and Sustainability Model (PRISM) to “shine light on multilevel context” and solve real world problems, such as advancing health equity, in a meaningful and sustained way (Stange). We round out the *Introduction* with two papers summarizing goals, resources, benefits of application, and future directions for RE-AIM by members of the National RE-AIM Workgroup (Smith and Harden, Harden et al.). These papers, along with the RE-AIM website (www.re-aim.org), provides readers with current definitions, examples, and resources for applying RE-AIM.

The second and third sections of the Research Topic are the *Use of RE-AIM in Community Settings* and the *Use of RE-AIM in Clinical Settings*, respectively. Papers in these sections include international studies focused on physical activity promotion in Brazil (Benedetti et al.) and work environment improvements in Denmark (Munch et al.). There are exceptional exemplars across these sections (Balis and Strayer; Balis et al.; Ball et al.; Wilcox et al.) to guide readers about the application of RE-AIM when planning, implementing, and/or evaluating specific interventions intended to improve effectiveness on participant health outcomes. There are also some thoughtful manuscripts illustrating qualitative and mixed methods approaches, and one applying RE-AIM in an iterative fashion to support ongoing improvements in implementation (Ball et al.; Kwan et al.; Glasgow et al.; Prusaczyk et al.). Finally, these sections conclude with an informative article about how to integrate an additional implementation science theory with RE-AIM constructs (King et al.).

Our Research Topic concludes with a collection of papers that reflect *Emerging Directions in the Application of RE-AIM*. Two studies and a commentary, focus on applications of RE-AIM (Baumann) in the areas of environmental health (Quinn et al.) and health policy (Toyserkani et al.). They provide interesting and novel adaptations that offer opportunities for replication and further evolution of the framework. This section, and the entire Research Topic, concludes with two papers that provide extensions to RE-AIM. One integrates Proctor's et al. conceptualization of implementation outcomes (Reilly et al.), and the other integrates PRISM and RE-AIM factors to advance sustainability and health equity (Shelton et al.). The papers in this section are strong examples of how RE-AIM can be conceptualized, re-conceptualized, and expanded to address and resolve. As called out in the Introductory commentary by Stange, these papers astutely address the wicked problems affecting the health and equity in our society by taking the path that creates and supports a high, robust, and sustained level of public health around the world.

It is our hope that this collection of papers provides concrete examples and guidance about how RE-AIM can be used to advance science and improve the public health impact in our communities, both nationally and internationally.

## Author Contributions

All authors contributed to the conceptualization of the manuscript and its content. All authors contributed to the full manuscript as well as reviewed and approved the final version of the manuscript.

## Conflict of Interest

The authors declare that the research was conducted in the absence of any commercial or financial relationships that could be construed as a potential conflict of interest.

